# Screening for red flags and modifying manual therapy techniques for pediatric patients

**DOI:** 10.3389/fped.2026.1751693

**Published:** 2026-03-05

**Authors:** Virginia K. Henderson, Jenifer Dice, Doug Dendy, Jean-Michel Brismée

**Affiliations:** 1 Physical Therapy, Texas A&M University Texarkana, Texarkana, TX, United States; 2 School of Physical Therapy, Texas Woman's University, Houston, TX, United States; 3 Department of Rehabilitation Sciences, Texas Tech University Health Science Center, Lubbock, TX, United States

**Keywords:** mobilization, physical therapy, physiotherapy, PUMT, red flags

## Abstract

**Objective:**

This mini-review synthesizes evidence for safe and effective pediatric joint mobilization, discussing red flags, offering clinical recommendations, and addressing current research limitations.

**Methods:**

A PubMed and PEDro search, supplemented by foundational pediatric musculoskeletal resources, identified peer-reviewed articles and guidelines, which experts collaboratively evaluated.

**Results:**

Pediatric musculoskeletal systems, with open growth plates and lower bone mineral density, are vulnerable to shear forces. Safe force thresholds remain undefined. Therefore, thorough history taking, systematic red flag screening, and adherence to International Federation of Manual and Musculoskeletal Physical Therapists guidelines are crucial.

**Discussion:**

The refined Pediatric Utilization of Manual Therapy tool (PUMT-2, combined with the proposed modified three-tier red flag framework, supports individualized, developmentally appropriate assessment and care. Future research should establish force guidelines and age-based technique modifications, alongside specialized clinician training to address pediatric-specific needs.

## Introduction

The principles of musculoskeletal rehabilitation apply across the lifespan. However, treating pediatric patients with joint mobilization requires a nuanced understanding of developmental anatomy, clinical decision-making, and evolving evidence (1). The treatment goals for both pediatric and adult populations commonly include pain reduction, increased strength, improved range of motion (ROM), and increased functional performance (2). While a strong body of evidence supports the use of joint mobilization for neuromusculoskeletal impairments in adults research specific to pediatric patients with a neuromusculoskeletal condition remains limited (1, 3–5).

A systematic review of English language studies on pediatric spinal manipulation, conducted on MEDLINE, PubMed, CINAHL, Cochrane Central Register, PEDro, and Sports Discus, identified only four studies meeting inclusion criteria for spinal pain or headaches, leading to the conclusion that evidence remains insufficient to either support or refute its efficacy for children with spinal neuromusculoskeletal conditions (5). A 2019 systematic review by Parnell Prevost et al. (6) included fifty studies and reported no serious adverse effects occurred when appropriately trained clinicians performed joint mobilizations in pediatric patients. The authors emphasized the need for more rigorous clinical trials (6). Similarly, Corso et al. (7) concluded that more research is needed on pediatric manual therapy, including mobilizations and spinal manipulation, in children under 10 but that current report of adverse events is rare (7).

The Limited pediatric literature available suggests potential benefits of joint mobilization when applied judiciously. Pediatric case-based evidence suggests that low- to moderate-grade joint mobilization, including treatment of involved and adjacent segments, may reduce pain and support functional participation in children with inflammatory or nonspecific musculoskeletal conditions (8, 9). Higher level evidence from an international randomized controlled trial demonstrated that targeted grade III ankle mobilizations in children with cerebral palsy improved dorsiflexion range of motion, balance, and gait velocity compared to controls (10).

Safety remains a central concern in pediatric manual therapy. Evidence emphasizes thorough history-taking and clinical examination to identify conditions requiring modification or avoidance of joint mobilization (11). In 2024, the International Federation of Orthopaedic Manipulative Physical Therapists (IFOMPT) published age-based guidance for spinal mobilization and manipulation in children, while the Pediatric Utilization of Manual Therapy (PUMT) decision tool was created to encourage clinicians to consider movement goals, red flags, pain phenotype, impairments, and multimodal follow-up (12, 13).

This review synthesizes evidence relevant to the safe and effective use of pediatric joint mobilization, discussing red flags, offering clinical recommendations while highlighting current research limitations. A mini-review design was selected to contextualize a focused body of literature examining the risks, safety considerations, and alternatives to joint mobilization in pediatric patients with a neuromusculoskeletal condition. Given the limited volume of pediatric-specific studies and the heterogeneity of study designs, this approach was chosen to provide a direct and concise synthesis of literature to identify critical knowledge gaps and suggest direction for future research (14). This method integrates expert perspective with primary research in a way that is accessible to practitioners and supports clinically meaningful interpretation and knowledge translation that might not be achievable through a systematic review (14). Therefore, this mini-review aimed to identify and synthesize evidence relevant to the safe and effective use of pediatric joint mobilization, discuss pediatric red flags, provide clinical recommendations, and highlight current research limitations.

## Methods

This mini review synthesizes current evidence and expert perspectives on manual therapy, and pediatric physical therapy (14, 15). The literature search focused on identifying relevant peer-reviewed articles and foundational resources addressing human development, pediatric musculoskeletal conditions, manual therapy, and clinical decision-making for pediatric patients with a neuromusculoskeletal condition (4, 12, 13, 16).

A targeted search of the PubMed and PEDro databases was conducted to identify research articles, reviews, and clinical guidelines relevant to the topic. Search terms included combinations of keywords such as *pediatrics, manual therapy, joint mobilization, contraindications, red flags*, and *musculoskeletal development.* In addition to peer-reviewed literature, content was drawn from *Campbell's Physical Therapy for Children* (6th ed.), a widely recognized textbook in pediatric physical therapy education. When information was extracted from Campbell's, efforts were made to locate and review the original research sources cited within the textbook to ensure the use of primary evidence whenever possible.

To enhance the clinical relevance and applicability of this review, an interdisciplinary group of content experts collaborated throughout the process. This group included orthopedic and manual therapy fellows, experienced pediatric physical therapists, and faculty members specializing in pediatrics from two Doctor of Physical Therapy (DPT) programs. Their collective expertise guided the interpretation of the literature, provided practical insights, and informed the development of recommendations for clinical practice.

A formal systematic screening process was not employed; rather, the aim was to provide a comprehensive, clinically meaningful synthesis of current knowledge to support safe and effective clinical considerations for the evaluation and management of pediatric patients with neuromusculoskeletal conditions and offer recommendations to guide future research.

### Considerations for manual therapy applications for the pediatric population

#### Growth plates

One of the most critical distinctions between pediatric and adult skeletal systems is that developing bones retain open growth plates. These epiphyseal plates drive longitudinal bone growth through endochondral ossification and respond to both hormonal and mechanical influences according to the mechanostat theory. While distraction forces can facilitate growth, excessive compression can inhibit it (17). However, clinicians still lack clear evidence on how manual joint forces affect growth plates.

Landing with an extended knee increases ground reaction forces, which when combined with poor landing mechanics raises the risk of growth plate injury (18). Joint forces applied through manipulation under anesthesia result in a breakdown of adhesions without growth plate damage (19). Clinicians need additional research to define safe force thresholds for manual therapy techniques in the presence of open growth plates.

Animal studies and biomechanical modeling in species such as rats and rabbits have shown that growth plates are particularly vulnerable to shear and compression forces (20, 21). This understanding of biomechanical susceptibilities, especially the elevated risk posed by shear stress, offers critical insight for guiding safe clinical practices. Furthermore, Ménard et al. found that traction forces may promote bone remodeling, with increased mineralization and new bone formation observed following the removal of both static and dynamic compression in rats (22). Notably, the physiological loading conditions applied in their study did not damage the growth plates. Because cartilage has lower shear and torsional tolerance than mature bone, manual and high-impact interventions should be graded according to the maturity of the ossification centers during skeletal growth (23).

#### Pediatric bone mineral density

A second important consideration is how manual forces affect bones with reduced bone mineral density (BMD). There are several scenarios in which pediatric bone density could be compromised. During adolescence, bone length increases faster than the body adds cortical bone density. This places the child at higher risk for fracture (16). Clinicians should account for both the patient's developmental stage and medical diagnosis when evaluating BMD risk.

Some conditions, such as osteogenesis imperfecta, directly compromise bone density, while other chronic illnesses can indirectly reduce BMD by impairing nutrition, physical activity, or hormonal regulation. These conditions include, but are not limited to, hemophilia, human immunodeficiency virus (HIV), myelodysplasia, spinal cord injury, juvenile idiopathic arthritis, childhood cancer, cerebral palsy (GMFM IV or V), arthrogryposis, myelomeningocele, scoliosis, celiac disease, and inflammatory bowel disease (24–35). Furthermore, down syndrome presents with low bone mineral density due to a combination of genetic and lifestyle factors (36).

Bone mineral density (BMD) is also a concern for children undergoing surgery. One study examining postoperative BMD following lower extremity surgery and four weeks of non-weight bearing reported an average BMD loss of 16.5%, with some patients experiencing losses as high as 34%. In adults, this degree of bone loss has been associated with a twofold increase in fracture risk (37). These findings highlight the importance of interventions that support BMD recovery while exercising clinical caution in children with diagnoses that increase fracture susceptibility.

Finally, children are at a higher risk for fracture following corticosteroid use. A 15-year cohort study found that children with asthma had an increased risk of fractures (aOR 1.22, 95% CI 1.11–1.35), with the highest risk observed in those with recent inhaled corticosteroid (ICS) use (aOR 10.77, 95% CI 10.28–11.28). Use of ICS, systemic corticosteroids, and daily prednisone were all significantly associated with fracture risk (38). A 6-year multicenter cohort study demonstrated that vertebral fractures are the hallmark of pediatric glucocorticoid-induced osteoporosis (GIO), occurring early in treatment and often asymptomatically, necessitating routine monitoring (39). Finally, a large population-based study found that ≥4 courses of corticosteroid use significantly increased overall fracture risk (aOR 1.32, 95% CI 1.03–1.69), with humerus fractures showing the greatest elevation (aOR 2.17, 95% CI 1.01–4.67). Higher daily doses (≥30 mg prednisolone) were also linked to increased risk (aOR 1.24, 95% CI 1.00–1.52). Importantly, children who discontinued corticosteroid use had fracture risks comparable to controls, highlighting that cumulative exposure is the primary determinant of skeletal vulnerability (40).

Clinical judgement is needed in determining appropriate manual therapy forces for pediatric patients as there remains a lack of universally established, standardized guidelines specific to force thresholds in this population (11). Bone mineral density is one component of clinical judgment and should be considered in conjunction with differences in children's tensile strength. This is necessary to define the limit at which pediatric manual therapy can be applied and avoid tissue damage (41). Furthermore, the child's communication ability, weight, and neurologic development are also necessary components of clinical judgement (41, 42). Although joint mobilization forces have been quantified in adult studies, pediatric-specific research remains limited and must account for BMD and the additional unique factors outlined above (41, 43, 44).

#### Ligamentous laxity

A third area of consideration is pediatric ligamentous laxity present in developing joints. Ligamentous laxity impacts joint stability and predisposes younger individuals to specific musculoskeletal injuries such as nursemaid's elbow (45). This condition, formally known as radial head subluxation, arises from the displacement of the radial head from the annular ligament, often due to a sudden longitudinal pull on the forearm (46). Manual therapy techniques that involve traction or gapping at areas of ligamentous laxity should be reconsidered in light of the decrease in rigidity of pediatric ligaments and incomplete ossification contributing to vulnerability, particularly at the annular ligament (46).

### Red flag identification and categorization

Clinicians must remain vigilant for signs warranting referral or indicating complex underlying conditions. The red flag chart in the PUMT tool article provides guidance by sorting red flags into three categories. It has been updated to reflect the findings of this review (Table 1) (13).

**Table 1:** Pediatric Red flags: to consider before initiating joint mobilizations.

Category	Description	Examples/Indicators
Category I—Immediate Medical Attention/Contraindication	Require immediate referral; do not perform joint mobilizations.	Active diagnosis at treatment joint: • Current cancer diagnosis • DislocationFracture • Fused joint • Legg-Calvé-Perthes • Osteogenesis Imperfecta • Osteoporosis • Slipped Capital Femoral Epiphysis Neurological status changes: • Bowel/bladder changes • Loss of developmental milestones • New neurological deficit • New onset strabismus • Progressive proximal weakness Serious constitutional signs: • Bulging/sunken fontanelle • Convulsions • Cyanosis • Dyspnea • Widespread loss of power
Category II—Further Questioning/Examination	May proceed after careful assessment; look for clusters of symptoms and evaluate severity.	Cancer concern: • Bone pain • History of cancer • Night pain • Unexplained bruising • Unrelenting pain • Concern for abuse—Injury with incongruent history Constitutional signs: • Acute weight loss (>5% body weight) • Fecal blood • Fever • Malaise • Persistent diarrhea • Persistent headache • Persistent vomiting • Signs of dehydration Other diagnostic indicators: • Hot, swollen, tender joints • Persistent inconsolable crying • Recent trauma • Unable to bear weight
Category III—Further Testing/Clinical Reasoning	Proceed only after additional evaluation or consultation; use clinical judgment.	Active diagnosis: • Arthrogryposis • Dystonia • Ehlers-Danlos Communication limitations: • Child too young to communicate adverse symptoms • Child too young to give consent Concern for osteoporosis: • Due to long-term non-weight bearing • Due to diagnosis or developmental stage Concern for region-specific ligamentous laxity: • Cervical spine • Annular ligament at proximal radioulnar joint • Prolonged corticosteroid use

Category I identifies situations needing urgent medical attention (e.g., malignancy, fracture, dislocation). Category II highlights factors requiring additional assessment, like previous cancer history or pronounced scoliosis. Category III includes areas demanding nuanced clinical judgment, such as challenges with communication or non-weight-bearing status, that are not absolute contraindications but require caution and individualized assessment. This tiered framework is valuable in pediatrics, as not all red flags prohibit joint mobilization. For example, gentle mobilization may be appropriate in children with Ehlers-Danlos when used judiciously to address pain or locking, despite inherent hypermobility (47, 48). Likewise, clubfoot correction in infants safely uses mobilization as part of the Ponseti method, supported by substantial literature (49–51). Importantly, these cases require strong clinical reasoning and alignment with evidence and patient factors (13).

### Clinical implications- integrating joint facilitations with joint mobilizations

Joint manipulation represents the most aggressive form of manual therapy. Reports of serious adverse events in pediatric patients with a neuromusculoskeletal condition following manual therapy most commonly involve missed underlying pathology or joint manipulation techniques involving spinal extension and rotation. Specifically, the literature review by Todd et al. (11) identified adverse events associated with mobilization performed by physical therapists, including fractures and a subarachnoid hemorrhage linked to the Vojta technique, which incorporated manipulation with electrical stimulation. Reported adverse effects from chiropractic and osteopathic interventions included crying, vomiting, fractures, headaches, neck stiffness, and irritability (6, 11). IFOMPT recommends against cervical and lumbar spinal manipulation in children aged 0–12 years but states that spinal manipulation may be appropriate in adolescents aged 13–18 with sound clinical reasoning (12).

In contrast to high velocity manipulation, joint mobilization is graded and grounded in arthrokinematics principles and typically performed at mid- or end-range, depending on the clinicians' therapeutic objectives (52). In the pediatric population, a child is less likely to cooperate with ideal positioning for joint mobilization and position adjustments will likely be necessary. When positional accommodations are made, it is important that the therapist maintains correct directional application and continues to stabilize other joints to reduce accessory motion. Skeletal development will impact the angle and force of the mobilization (52, 53). While the safety and benefits of joint mobilization are well documented in adult populations, evidence supporting its safety in pediatric patients with compromised bone density or complex diagnoses remains lacking in the literature (3, 4, 13).

**Figure 1:**
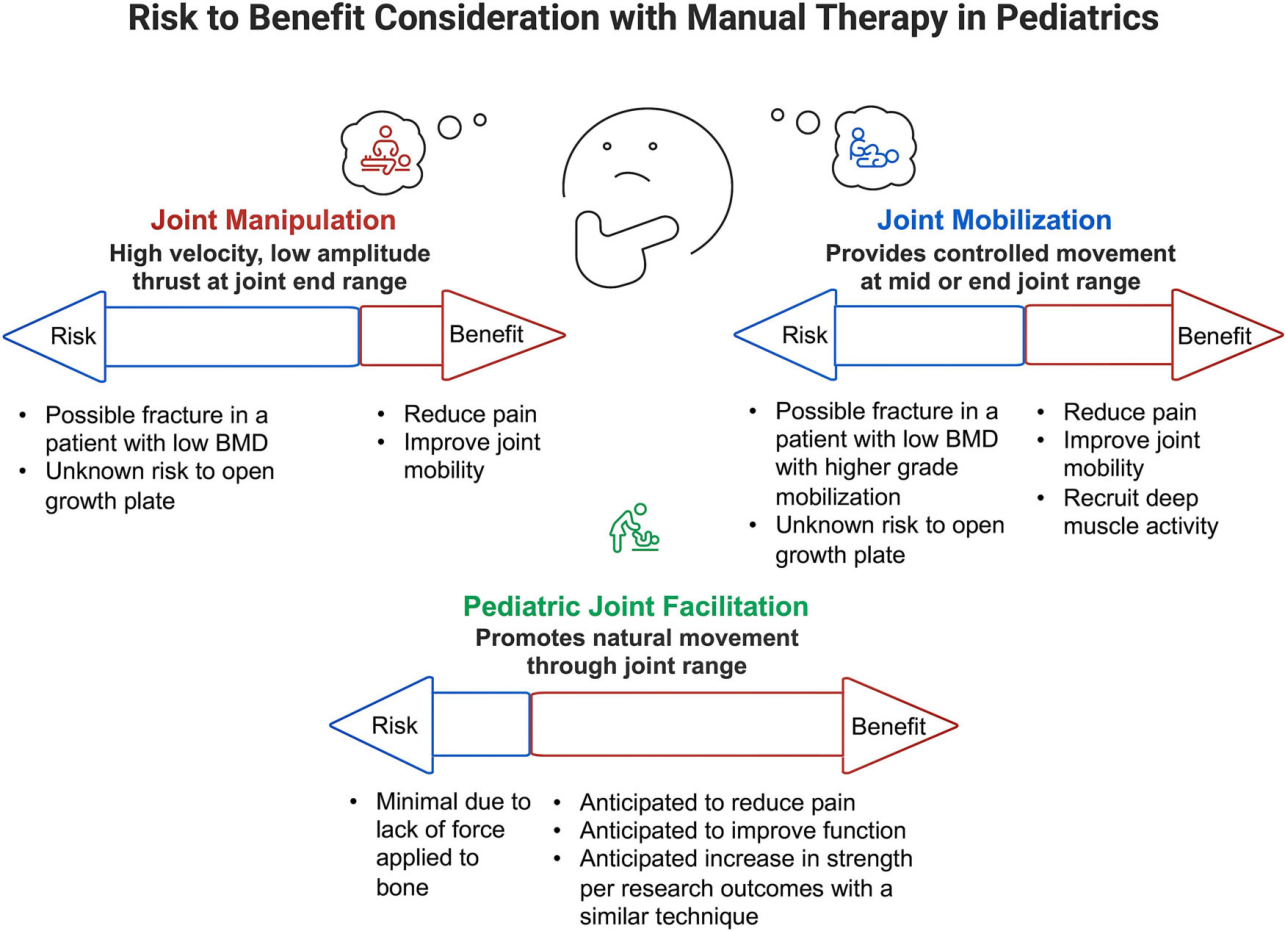
The risk/benefit comparison of joint manipulation, joint mobilization, and pediatric joint facilitation (3, 52, 54, 60).

When treating pediatric patients who may benefit from joint-level intervention but present with red flags that warrant caution with joint manipulation or mobilization, clinicians might consider a joint-facilitation technique (Figure 1). This allows for direct joint input paired with passive and active movement that employs low-force facilitation within physiological limits and positions aligned with movement and postural goals. Such techniques aim to improve joint position, postural control, movement mechanics, and functional performance in children with neurological and/or orthopedic conditions while considering tensile strength and BMD. This facilitated movement allows for the benefit of joint mobility without the risk of isolated and sustained load.

Two techniques that promote joint facilitation, that have been studied in adults, Neuromuscular Joint Facilitation (NJF) and Movement with Mobilization (MWM). Neuromuscular Joint Facilitation integrates joint facilitation with proprioceptive neuromuscular facilitation (PNF) techniques (54). Studies on NJF have demonstrated improvements in joint mobility, with additional benefits in balance, pain reduction, muscle activation, joint position sense, and strength—findings that align with outcomes reported for joint mobilization alone (54–57). These results support the therapeutic value of combining movement and mobility interventions at the joint level.

Movement With Mobilization, by contrast, is designed to correct joint positional faults through sustained accessory mobilization applied concurrently with a specific active movement. This technique promotes immediate pain relief and functional improvement through patient-directed, therapist-facilitated motion. A systematic review by Stathopoulos et al. supports the efficacy of MWM in reducing both pain and disability across peripheral joints (58). These findings reinforce the value of combining mobility and movement in treatment.

### Limitations of the review

This review has limitations that should be acknowledged. First, the available research specifically addressing pediatric joint mobilization is limited in both quantity and scope. Much of the current understanding is extrapolated from adult studies or based on clinical expertise rather than high-quality pediatric-specific research. As a result, the recommendations presented rely in part on expert opinion and clinical reasoning rather than a robust, evidence-based foundation.

Additionally, while a targeted literature search was conducted, this review does not constitute a systematic review, and there is a possibility that relevant studies may have been missed. A scoping review would be another methodological alternative to broaden the range of literature available and enhance the understanding of the subject. Furthermore, information drawn from widely used educational resources, such as *Campbell’s Physical Therapy for Children*, was included to provide context; however, some of this material references studies that may be older or have methodological limitations.

Finally, the interpretation of the literature and development of clinical recommendations were informed by expert input from pediatric physical therapists, orthopedic and manual therapy fellows, and academic faculty. While this enhances the clinical relevance of the review, it also introduces the potential for bias based on the perspectives and experiences of those involved (59).

Future research with pediatric-specific study designs is essential to better define the safety, efficacy, and clinical guidelines for joint mobilization in children.

## Conclusions and recommendations

The safe and effective application of joint mobilization in pediatric patients with a neuromusculoskeletal condition requires careful clinical reasoning, thorough screening, and adherence to current guidelines. Based on the available evidence and expert consensus, the following recommendations are proposed to support best practice:

Clinicians should incorporate the three-tier red flag screening chart (Table 1) to systematically identify conditions that may necessitate outside referral, contraindicate joint mobilization, or require caution with joint mobilization.
Providers should follow established guidelines from IFOMPT, which discourage spinal manipulation in infants and young children and emphasize caution in adolescents.
A thorough clinical history and understanding of BMD risks is critical for identifying red flags that may not be evident during physical examination.

Future advancements in pediatric manual therapy should prioritize both safety and developmental appropriateness by considering techniques that activate joint receptors, promote motor control, and promote positioning without applying excessive force through immature skeletal structures.

Further research is needed to:

Define the role of end-range mobilization in specific pediatric patients with a neuromusculoskeletal condition with habitual positioning or movement impairments.
Establish consensus on pediatric-specific red-flag screening tools.
Evaluate the effectiveness of joint facilitation techniques compared to traditional pediatric treatment.
Investigate the value of modifying techniques for 0–2 year-olds, 2–12 year-olds, and 13–19 year-olds. What needs and challenges arise for each age group, considering body size, development, and communication ability?
Establish guidelines for appropriate force application with pediatric joints.

Additionally, there is a clear need for specialized training to enhance clinician competence in recognizing pediatric-specific concerns and appropriately applying manual therapy techniques.
